# Learner-Level Psychological Factors Impact Feedback Recipience in Medical Education

**DOI:** 10.1007/s40670-025-02448-y

**Published:** 2025-07-04

**Authors:** Lynnea M. Mills, Pooja Lalchandani, Olle ten Cate, Christy Boscardin, Patricia S. O’Sullivan

**Affiliations:** 1 Department of Medicine, University of California, San Francisco, USA; 2 Department of Medical Education, University Medical Center Utrecht, Utrecht, Netherlands; 3 Departments of Anesthesia and Medicine, University of California, San Francisco, USA; 4 Departments of Medicine and Surgery, University of California, San Francisco, USA

**Keywords:** Feedback, Emotion, Feedback orientation, Feedback retention

## Abstract

**Purpose:**

Gaps exist in the literature concerning psychological factors impacting medical learners’ receptivity to feedback. Learners’ orientation toward feedback and their emotions during feedback are likely to influence their recall of feedback and therefore their ability to act upon it. Better understanding these constructs and relationships could improve feedback processes.

**Materials and Methods:**

We conducted a feedback simulation study with a pre-simulation measure of feedback orientation (FO), an in-simulation measure of emotions experienced, and a post-simulation measure of recall. Participants were third- and fourth-year medical students at one US medical school.

**Results:**

Twenty-two students participated. FO scores were higher than in prior work in medical education. Emotions during feedback were mixed but mostly positively valenced and mostly activating. Students recalled, on average, 0.77 of 2 specific reinforcing feedback points and 1.0 of 2 constructive points. There were small correlations among the constructs; specifically, positive emotional valence was slightly negatively correlated with recall.

**Conclusions:**

FO and emotion during feedback are two factors that may influence learners’ retention of feedback. The results indicate these factors are complex and related, requiring further studies. Additionally, our results call for work to expand on and improve psychological measurement tools when used with medical learners.

## Introduction

Research has revealed much about how medical learners respond to and incorporate feedback, but there remain key gaps in the literature. Feedback models have moved from unidirectional purveyance of information to contemporary models emphasizing the role of the feedback-receiver in the process [1, 2]. While these models consider how learners approach, process, and incorporate feedback, research has not extensively examined how individual learner factors influence feedback uptake, particularly psychological factors such as learners’ beliefs about and attitudes toward feedback, and the emotions that arise for them during feedback. Understanding more about these factors and how they impact learners’ ability to retain and uptake feedback would likely improve feedback processes in medical education.

### Feedback Models

**Fig. 1:**
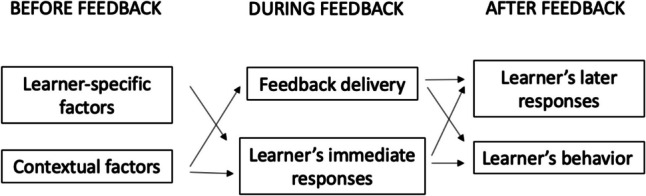
General model of components of the feedback process, representing a synthesis of feedback models that incorporate learner-level factors

Mostly grounded in general education and psychology literatures, current feedback frameworks generally emphasize specific discrete components or stages of the feedback process, which we outline broadly in Fig. 1, based on a review by Lipnevich and Panadero [1]. Before a feedback interaction, individuals bring personal factors to feedback in general, combined with contextual factors such as their relationship with the feedback-giver. These influence how the learner approaches individual feedback interactions. During feedback, the interaction involves components such as the delivery and the learner’s in-the-moment response. All these factors lead to appraising and processing the feedback, which may produce behavior change and performance improvement, in addition to responses like coping mechanisms.

There remain numerous gaps in our understanding of these factors and how these processes interrelate to create feedback responses. Demystifying any of these processes or relationships could allow medical educators to tailor learner-level approaches. We have identified three specific factors, among many, that merit further research. Regarding “before feedback,” previous work in medical education has highlighted feedback orientation (FO) [3]. FO is a learner’s receptivity to feedback in a durable way; it is considered a quasi-trait because it can change somewhat over the long term but is generally stable across contexts for a given learner. FO affects performance outcomes [4], but it is unclear by what mechanisms. We suspect FO may, in part, impact outcomes by affecting in-the-moment responses to feedback.

In the “during feedback” stage, published models account for both cognitive and affective (related to emotion, motivation, and other psychological domains) reactions in learners [5, 6]. Affect, specifically emotion, is a major factor in how learners respond to feedback. Researchers have shown that emotion affects the learning process through neural mechanisms [7]; emotions experienced during feedback may directly impact learning that results from feedback. Indeed, research in psychology demonstrates that emotion mediates the relationship between feedback and aspects of performance change [8]. However, it is unclear how learners’ emotions might interact with FO to produce a learner’s uptake of feedback.

Finally, “after feedback” concerns the use of feedback for performance improvement. Retention of feedback is a key cognitive process enabling use of feedback. Across disciplines and learner levels, students generally retain small amounts of their teachers’ feedback [9–11]. Most noteworthy are studies investigating learners’ recall of reinforcing (“positive,” what was done well) vs constructive (“negative,” what to improve on) feedback, with contradictory results [12–14].

In considering the work relevant to the “before,” “during,” and “after” stages of feedback, we note that many studies do not examine the impact of orientation and emotion on feedback retention. Psychology and neuroscience studies have explored the contexts, mechanisms, and degrees to which emotion impacts memory [e.g., 15, 16] and have shown that emotion affects how people recall big and small events. The impact of an emotion on retention is generally believed to depend on two factors: the emotion’s valence (positive or negative) and its level of arousal [17]. Retention is often enhanced by the presence of a positive or negative emotion [18], with some research indicating that negative emotions are more associated with lasting memory [17]. Through an independent neural process, the level of arousal also impacts retention, with more activating/arousing emotions provoking stronger memories [19]. Arousal may have less of an impact than valence, however, because valence persists longer as part of individuals’ experience [20]. The dimensions of valence and arousal are complex, but viewing them as bipolar, that is, on a spectrum from positive to negative and from activating to deactivating, and isolating the effects of these two dimensions, has enabled research like that outlined above, which has offered important insights about how emotion works in real individuals. While some authors have begun discussing the important role of emotion in learning in medical education, via memory and other pathways [e.g. 7], this area is ripe for further exploration.

**Fig. 2:**
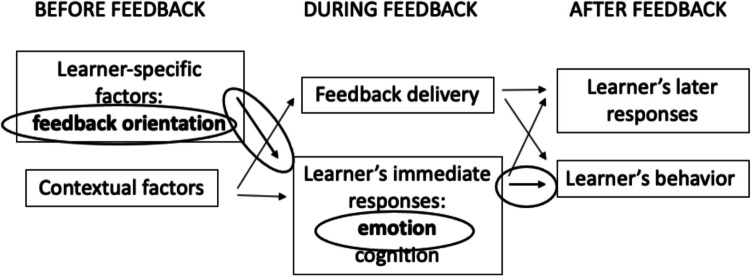
General model of the feedback process with components under exploration in this study circled

We suspect that FO and emotional responses to feedback work together to impact feedback retention and thereby how learners use feedback. Figure 2 builds on the initial feedback model to highlight key constructs and relationships that are the focus of the current study.

At this stage, the health professions education literature has very limited discussion of the different psychological factors at work in the feedback process and how we can best study them going forward. We designed an exploratory study to provide foundational information about FO, emotions, and recall, as well as the relationships among them. We anticipated this study would provide groundwork for further explorations of how emotion and orientation interact with and affect learners’ feedback receptivity.

## Materials and Methods

### Study Design

**Fig. 3:**
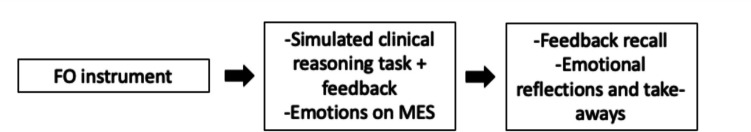
Overview of the activities for participants in each phase of the study

This is a descriptive study of participants’ psychological experience during a feedback simulation. It includes pre- and post-simulation data to inform our understanding of the relationships among the elements of FO, emotion, and retention. (See Fig. 3 for an overview of the study phases.) We felt a simulation enabled us best to lay the foundation for emotions and feedback.

### Participants and Setting

Participants were medical students in the two senior classes (MS3 and MS4) at one US medical school. These students had experience receiving feedback on clinical work. We did not collect participant demographic data.

### Simulation Activity

During fall 2024, each participant attended a 1:1 virtual interaction with a member of the research team who was not known to them or involved in their assessments, and who did not have access to their other data from the study. In this interaction, the researcher presented written details of a clinical case of a patient with acute altered mental status (minimally adapted from a published case [21]). The case spans multiple specialties and has a broad differential. The researcher asked the student to talk through their differential diagnosis as if they were on an inpatient service, about to admit the patient from the Emergency Department. Immediately afterward, the researcher provided feedback on the participant’s reasoning. The feedback was individualized to each participant but was standardized to include five discrete components: one general reinforcing feedback point (e.g., “Great job thinking on your feet”), two pieces of specific reinforcing feedback (e.g., “I really liked how you included potential medication effects in your differential”), and two pieces of constructive/modifying feedback (e.g., “also consider metabolic abnormalities that could lead to confusion.”). As soon as the feedback experience ended, students were asked to indicate the emotions they felt when receiving the feedback.

### Instruments

#### Feedback Orientation

FO was measured using a 20-item FO instrument with reliability (Cronbach’s alpha 0.86) [22], and additional validity [e.g., 23], evidence. Participants completed the FO instrument online (Qualtrics, Seattle, WA). Each item is rated on a 1 (low FO)–5 (high FO) Likert scale and total FO score is obtained by averaging all item scores. Sample items include “feedback contributes to my success at work” and “I feel self-assured when dealing with feedback.”

#### Emotions

**Fig. 4:**
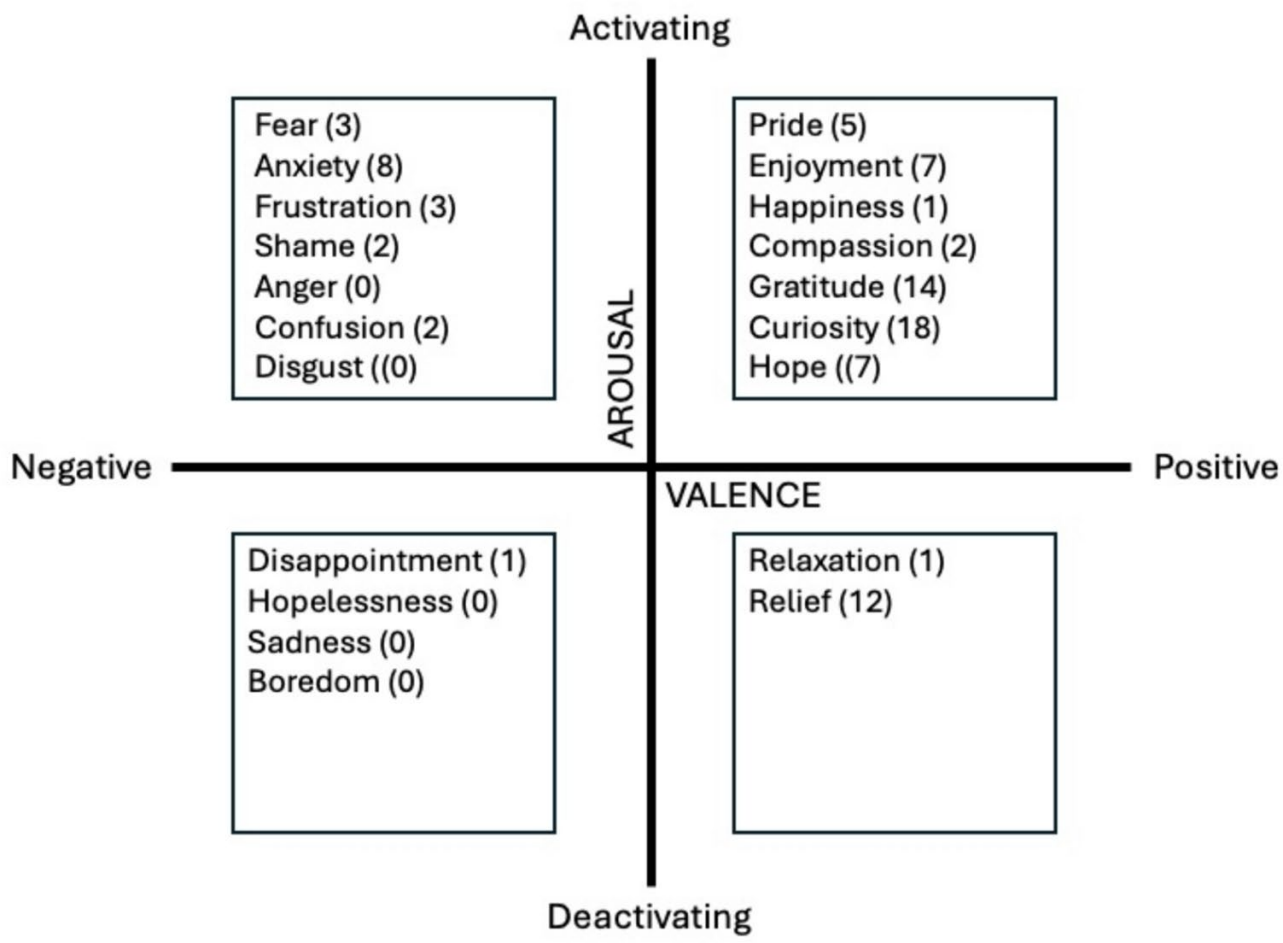
Emotions endorsed by participants, arranged by MES quadrant on the orthogonal valence-arousal bipolar scales; numbers in parentheses represent the number of participants who endorsed each emotion

Students completed the Medical Emotions Scale (MES), a multidimensional inventory of medical learners’ emotions, with validity support through comparison with a theoretical model of expected emotions and with interview findings, and outcomes correlations in learning environments [24]. This scale includes 18 emotions grouped based on valence (positive vs negative) and arousal (activating vs deactivating). (The MES also includes two neutrally valenced emotions that we excluded, to focus on valence.) These emotions include fear, disappointment, and enjoyment; the full list of MES emotions used in this study is included in the results, in Fig. 4. Students selected as many emotions as were applicable to them. We recorded the number of positive activating, positive deactivating, negative activating, and negative deactivating emotions for each participant.

#### Recall

Two to three weeks after the simulation, participants were asked to write all the feedback they recalled receiving during the simulation. This recalled feedback was categorized as general reinforcing (one item), specific reinforcing (two items) and constructive (two items), and points were recorded only if statements reflected the actual feedback given.

#### Perceptions

To affirm and expand on MES data regarding their emotions, students responded to prompts at 2–3 weeks after the simulation. These were as follows: “Please use this box to describe anything you felt about your feedback interaction during or since that experience. This can include elaborating on the emotions you indicated in the post-interaction survey or sharing anything that might not have been captured in that checklist”; and “What, if anything, do you think you have taken or will take away from the feedback interaction?”

#### Procedures

We sent email solicitations to class listservs and enrolled students who replied. We did additional targeted outreach via email and in person. All data were collected online using the Qualtrics platform. Students completed the FO scale pre-simulation, MES immediately following the simulation, and the final survey 2–3 weeks following the simulation. Participants received $25 in gift cards for completing all parts of the study. The simulation interaction with each participant was conducted virtually (Zoom, San Jose, CA); interactions were recorded and transcribed in real-time by Zoom artificial intelligence software. Transcripts were reviewed and corrected as needed by the researcher immediately after the interaction. De-identified transcripts were then uploaded to a secure server for analysis and for other team members to ensure the feedback-giver was consistently adhering to the study protocol and to verify the recalled feedback. The school’s institutional review board deemed the study exempt (IRB reference number 397085).

#### Analysis

We calculated descriptive statistics for FO scores, emotions experienced, and feedback points recalled. We organized participants’ selected emotions on the MES based on valence and arousal. Two authors tallied recalled feedback points for each participant and whether they were reinforcing or constructive, and compared these to the transcripts to check for accuracy of recall. Two authors also performed content analysis of students’ qualitative comments on their emotions and takeaways. We used Spearman’s rho to examine relationships among FO score, emotions, and feedback recall. Spearman’s rho is a nonparametric measure of rank correlation, best utilized for ordinal data, such as in our study, where linearity cannot be assumed. Based on other work in health professions, we interpreted the findings as follows: Spearman’s rho < 0.2 = negligible, 0.2–0.4 = small/weak, 0.4–0.6 = moderate, > 0.6 = large/strong [25].

## Results

Twenty-two students were recruited and participated in all components of the study. The simulation interaction lasted approximately 10 min (SD = 2.90; range = 5 min 46 s–18 min 20 s), and the feedback portion lasted on average 1 min, 37 s (SD = 0.47; range = 0.56 s–2 min 41 s). Overall, FO scores ranged from 3.6 to 4.9, with a mean of 4.3 (SD = 0.38; scale 1–5). (Mean from the prior study on medical learners was 4.08 [3].) Participants indicated experiencing a mix of emotions during the feedback interaction, with a range of two to seven emotions per participant. Nineteen students experienced more positively valenced emotions than negative ones; three students indicated an equal number of positive and negative emotions, and no student indicated experiencing more negative emotions than positive ones. The most commonly experienced emotions were curiosity (18 participants), gratitude (14), and relief (12). Organized by quadrants of the MES, the emotions were mostly positive activating (54 total across participants), followed by negative activating (18) and positive deactivating (13). Only one student reported a negative deactivating emotion, disappointment. See Fig. 4 for endorsed emotions and Table 1 for a summary of the descriptive data.

**Table 1:** Descriptive data for feedback orientation and emotions

Metric	Mean	Standard deviation
Feedback orientation score (FO) (scale 1–5)	4.35	0.38
# positively valenced emotions (out of 9 possible on instrument)	3.05	1.00
# negatively valenced emotions (out of 11 possible on instrument)	0.86	1.04
# activating emotions (out of 14 possible on instrument)	2.90	1.54
# deactivating emotions (out of 6 possible on instrument)	0.63	0.58
# reinforcing feedback points recalled (out of 2 possible)	0.77	0.81
# constructive feedback points recalled (out of 2 possible)	1.00	0.69
Total # feedback points recalled (out of 5 possible)	1.77	1.15

Students recalled varying amounts of feedback from their interactions, with a mean of 1.77 (SD 1.15) and range of 0–3 (of 5) items correctly recalled. No student explicitly reported back the general reinforcing comment (e.g., “great job walking through the case”), but most (18) students recalled some of the specific reinforcing and/or constructive feedback points. Of those who recalled some points correctly, 8 participants recalled more constructive than reinforcing points, 4 recalled more reinforcing than constructive points, and 6 recalled an equal number of both. When students were scored as having recalled 0 feedback points, this was because they stated they did not remember or their response was not specific (e.g., “My preceptor started by telling me what I did well” without indicating what that feedback was).

In the qualitative responses, students primarily wrote positive comments. Participants shared that the interaction was “helpful,” or that they felt “grateful” or “validated.” They reiterated emotions from the MES such as anxiety, frustration, and relief, while adding synonyms like “nervous” or “stressed.” Four students explicitly conveyed a trajectory of initial negatively valenced emotions that turned more positive (i.e., anxiety giving way to relief) after completing the feedback interaction. Several cited relief upon hearing the feedback, and some commented on not recalling the exact feedback but remembering a pleasant experience.

In reflecting on their takeaways, students shared a mix of clinically focused and metacognitively focused comments. Twelve students’ main takeaway was a clinical pearl (e.g., to remember a particular item on the differential for altered mental status). For five, the takeaways related to how they received and processed the feedback. For example, one remarked on the difficulty of using feedback for growth instead of just a confirmation of being right. For all students who indicated a specific takeaway, the comments were positive.

Correlations help to describe associations between variables in this study. There were negligible correlations between FO scores and the valence and arousal of the emotions experienced, as well as between FO scores and feedback recall, with the exception of a small negative correlation between FO score and the amount of reinforcing feedback recalled. There were generally small correlations between positively valenced emotions and recall, which were negative for positive activating and positive for positive deactivating emotions. There were negligible correlations between activating emotions and recall. Correlations between deactivating emotions and recall were weakly positive. See Table 2 for correlation data.

**Table 2:** Spearman’s rho correlation matrix for quantitative data

	A	B	C	D	E	F	G	H	I	**J**	K	L
A	1	0.12	− 0.10	0.08	− 0.09	0.05	− 0.08	− 0.01	− 0.08	− 0.25*	0.18	− 0.10
B		1	N/A	N/A	N/A	N/A	N/A	N/A	N/A	− 0.27*	− 0.42**	− 0.44**
C			1	N/A	N/A	N/A	N/A	N/A	N/A	0.40*	0.25*	0.41**
D				1	N/A	N/A	N/A	N/A	N/A	− 0.10	− 0.31*	− 0.27*
E					1	N/A	N/A	N/A	N/A	− 0.03	0.07	0.08
F						1	N/A	N/A	N/A	− 0.22*	0	− 0.18
G							1	N/A	N/A	− 0.10	0.07	0.02
H								1	N/A	− 0.07	− 0.09	− 0.07
I									1	0.32*	0.25*	0.35*
J										1	N/A	N/A
K											1	N/A
L												1

Bolded results = non-negligible correlations. “N/A” indicates correlations not performed because they were not relevant to the study

*A*, FO score; *B*, # positive activating emotions; *C*, # positive deactivating emotions; *D*, total # positive emotions; *E*, # negative activating emotions; *F*, # negative deactivating emotions; *G*, total # negative emotions; *H*, total # activating emotions; *I*, total # deactivating emotions; *J*, # reinforcing points recalled correctly; *K*, # constructive points recalled correctly; *L*, total # points recalled correctly

*Small/weak correlation

**Moderate correlation

## Discussion

Twenty-two students participated in the simulation; overall, they had high feedback orientation (FO), experienced positive emotions during the simulation, and, at a later date, recalled some of the feedback they received. Most of the correlations we observed between variables were small or negligible. These results led us to conclude that the relationships explored in this study are more complex than our current level of understanding in health professions education research. Here, we convey our interpretation of the findings for the constructs at each stage of the feedback process and their relationships, to unpack the role of emotions in the uptake of feedback. While there are important limitations, the study reveals areas where future work could enhance the feedback experience.

In the “before feedback” stage, our participants’ FO scores were high. This study may have attracted participants with favorable views toward feedback, and this restricts the range of the correlation coefficients that could contribute to our results. The (albeit small) negative correlation between FO and recall for reinforcing feedback and positive correlation between FO and recall for constructive feedback suggest that learners with relatively high FO scores tend to focus on how they can improve. These results on recall raise questions on the type of feedback best retained by learners with lower FO scores, who are not well represented here. While there are data on how other psychological constructs such as self-esteem impact the recipience of reinforcing vs constructive feedback, probably largely related to concordance of the feedback with learners’ own self-images [26], these constructs are not the same as FO. We have limited data on how FO relates to learners’ processing of different types of feedback, and this requires further research.

The absence of correlation between FO and emotions during feedback, or between FO and overall feedback recall, is somewhat surprising. There are several possible explanations for this. One key explanation relates to context and another to methodology. In management science, FO was felt to lead to performance improvement via feedback-seeking behaviors [22]. Learners with higher FO were more likely to request feedback, which may have impacted factors such as how much feedback they were given. In our study, the learners were all given feedback automatically, which we find is common in medical education settings. Quite possibly, the construct of FO needs to be re-examined and modified to account for how learners respond to feedback they do not request.

Methodologically, the sample size here was small and the range on each measure was limited, suggesting a value in both replicating this study with a larger sample and carefully reviewing the instruments, which were fairly blunt. While the FOS and MES have good validity evidence, the combination of these constructs, along with a recall measure, which was limited to 5 points, may suggest the need for further development of these types of measures.

The results related to learners’ emotions in the “during feedback” stage revealed nuances related particularly to valence. First, there were many positively valenced emotions surrounding the interaction. The most common was curiosity, which is known to be positively correlated with motivation and improved mental health in medical students [27]; but, surprisingly, curiosity and other positive activating emotions did not seem to facilitate recall. We suspect that, while emotions like curiosity may stimulate motivation, they may need to be balanced with other factors or emotions for optimal effect on recall. For negatively valenced emotions, there was clustering in the “activating” category. These results offer more details than previously understood about learners’ specific emotions during feedback. Additionally, while data in higher education relate to the valence of emotions that learners experience when getting feedback after grades [28], our findings offer insights about emotions in the context of gradeless (formative) feedback. Notably, many of our participants experienced only positively valenced emotions, contradicting research indicating that constructive feedback provokes negatively valenced emotions [8]. This finding could be due to the lower stakes of the simulation experience, which did not provoke as much negative emotion, and merits further exploration in authentic settings.

Many of our participants, however, did endorse feeling both positively and negatively valenced emotions. This presence of “mixed emotions” is increasingly explored in psychology [29]. Our finding indicates that the valence component is more complex than implied by a bipolar scale. The MES is based on the circumplex model, which has undergone much study in psychology [e.g., 30, 31] and which regards valence and arousal as the two cardinal characteristics of emotion. However, some research is calling into question this model of emotions. Traditional approaches view valence as a single spectrum from positive to negative, which does not allow for holding both positive and negative emotions simultaneously [32]; this issue is unresolved in the research community [32]. Some researchers also question whether valence and arousal are sufficient to characterize emotions, with some including other dimensions such as origin (whether an emotion arises automatically or after processing) [33], duration [34], and frequency [35] as key characteristics. Our findings do suggest that aspects of emotion besides valence and arousal might be important. For example, the fact that multiple students commented on a trajectory from negative to more positive emotions implies duration, expectation, and relationships among emotions. We would argue that our findings support the need to explore more sophisticated concepts of emotions’ dimensionality.

Recall was slightly worse when participants experienced more positively valenced emotions. This supports evidence that negative emotions better stimulate memory [17]. However, this may be too simplistic a model: valence alone may not offer meaningful associations; instead, the interaction of valence and arousal (and possibly other dimensions) may be more relevant. This interaction finding supports the concept that initially drove the development of the circumplex model of emotion (as described above and used in the MES); the model was designed with the belief that valence and arousal do not operate independently [36]. Our data did show different correlations for the different valence × arousal interactions. We suspect that because of the small sample size and the limitations of the instruments, the study needs to be repeated to see if these are stable correlations. However, other work does suggest that this interaction of valence and arousal may be important. For example, positive deactivating emotion is known to facilitate memory, potentially by lowering cognitive load, in medical learners [37].

We have explored the “after feedback” construct of feedback recall through the correlation results described above, but the descriptive findings on recall highlight differential recall based on feedback type. Consistent with data from other fields [e.g., 38], our learners had suboptimal feedback recall and did not include the general reinforcing feedback message when asked what they recalled. This may be due to not viewing this message as “feedback,” which would corroborate data on the lack of utility of non-specific, compared to specific, feedback [e.g. 39]. Overall low feedback recall may relate to a variety of factors, including that learners may have focused on the specific reasoning task and the feedback may have felt peripheral to them. The differential retention of reinforcing and constructive feedback is important because it may indicate these two types of feedback affect learners differently. Both reinforcing [39] and constructive [40] feedback benefit learners, but it is unclear by what mechanism each affects performance improvement. Our data suggest that recall of feedback differs depending on whether the feedback is reinforcing or constructive and that this difference may be partially mediated by emotion.

There are several limitations of this study. The sample was small, due to the logistics of the multiple phases; the study likely attracted participants who were interested in and oriented toward feedback. We did not collect demographic data, and there could be meaningful differences in these constructs across identity groups. While the feedback-giver gave individualized feedback on each student’s performance, she standardized it into a specific number of reinforcing and constructive points. The duration or depth of the feedback exchange may not represent what is typical at some institutions. While this study explored the learner’s experience of feedback, the simulation did not allow for true dialogue between feedback-giver and feedback-receiver, which is recommended in the literature [41]. Finally, this study does not explore actual feedback uptake; we do not know how the students might have used this feedback to improve performance. However, we believe that students’ retention of feedback, combined with their reflections on the experience, can serve as a surrogate marker or precursor to feedback uptake.

## Conclusions

This study explores nuances of how different factors work together to produce a learner’s response to a given feedback interaction. Participants had high FO scores and mixed but primarily positive emotions during feedback. They recalled a portion of the feedback, preferentially constructive, and recall was weakly negatively associated with positive emotions. Our findings suggest that each component of the learner’s experience of feedback is highly complex but not unknowable. More broadly, they argue to continue studying these factors involved in feedback, and their interrelationships, as well as developing more robust and sophisticated measurement tools for these key constructs. This future work can tie into other research traditions and may inform debates about how learning occurs or how to bolster it.

## Declarations

No artificial intelligence was used in this study or preparation of the manuscript except as indicated in the text, to transcribe the simulation interactions.
